# Extracellular Polymeric Substances Acting as a Permeable Barrier Hinder the Lateral Transfer of Antibiotic Resistance Genes

**DOI:** 10.3389/fmicb.2019.00736

**Published:** 2019-04-17

**Authors:** Xiaojie Hu, Fuxing Kang, Bing Yang, Wei Zhang, Chao Qin, Yanzheng Gao

**Affiliations:** ^1^Institute of Organic Contaminant Control and Soil Remediation, College of Resources and Environmental Sciences, Nanjing Agricultural University, Nanjing, China; ^2^Environmental Science and Policy Program, Department of Plant, Soil and Microbial Sciences, Michigan State University, East Lansing, MI, United States

**Keywords:** extracellular polymeric substances, antibiotic resistance genes, lateral gene transfer, transformation, cell permeability, binding, model computation

## Abstract

Antibiotic resistance genes (ARGs) in bacteria are emerging contaminants as their proliferation in the environment poses significant threats to human health. It is well recognized that extracellular polymeric substances (EPS) can protect microorganisms against stress or damage from exogenous contaminants. However, it is not clear whether EPS could affect the lateral transfer of ARGs into bacteria, which is one of the major processes for the dissemination of ARGs. This study investigated the lateral transfer of ARGs carried by plasmids (pUC19, pHSG298, and pHSG396) into competent *Escherichia coli* cells with and without EPS. Transformant numbers and transformation efficiency for *E. coli* without EPS were up to 29 times of those with EPS at pH 7.0 in an aqueous system. The EPS removal further increased cell permeability in addition to the enhanced cell permeability by Ca^2+^, which could be responsible for the enhanced lateral transfer of ARGs. The fluorescence quenching experiments showed that EPS could strongly bind to plasmid DNA in the presence of Ca^2+^ and the binding strength (Log*K*_A_ = 10.65–15.80 L mol^-1^) between EPS and plasmids was positively correlated with the enhancement percentage of transformation efficiency resulting from the EPS removal. X-ray photoelectron spectroscopy (XPS) analyses and model computation further showed that Ca^2+^ could electrostatically bind with EPS mainly through the carboxyl group, hydroxyl group, and RC-O-CR in glucoside, thus bridging the plasmid and EPS. As a result, the binding of plasmids with EPS hindered the lateral transfer of plasmid-borne ARGs. This study improved our understanding on the function of EPS in controlling the fate and transport of ARGs on the molecular and cellular scales.

## Introduction

The discovery of antibiotics has saved millions of human lives and also made animal agriculture more productive and profitable. However, imprudent use of antibiotics has led to the widespread antibiotic resistance in bacterial populations, which poses significant threats to human health (Martínez, 2008; Amy et al., 2012; Mckenna, 2013; Modi et al., 2013). Antibiotic resistance genes (ARGs) are now considered as emerging contaminants (Amy et al., 2006). It is well known that both clonal expansion and lateral gene transfer (LGT) contribute to the spread of antibiotic-resistant bacteria (ARB) and ARGs. Compared to clonal expansion that passes genes from parent cells to offspring cells, LGT can transfer ARGs across organisms of the same or different species, which may substantially increase the risk of ARGs (Blahna et al., 2006). LGT occurs in the environment mainly through three mechanisms: transformation, transduction, and conjugation. For transformation to occur efficiently, bacterial cells need to acquire natural competence, a physiological state of cells essential to the uptake of extracellular DNA (Scrudato and Blokesch, 2012). It is also well established that many bacteria secrete an extracellular matrix that is complex but mainly composed of polysaccharides and proteins (about 75–95%; More et al., 2014; Gunn et al., 2016). This extracellular matrix is termed extracellular polymeric substances (EPS).

Extracellular polymeric substances usually serve as a barrier protecting cells from external environmental stress, such as extreme temperature (Aslam et al., 2012), extreme pH (Dogsa et al., 2005), and high salinity (Ismail et al., 2010). Additionally, EPS also helps bacteria cope with exogenous contaminants (e.g., heavy metals, nanoparticles, and antibiotics; Joshi and Juwarkar, 2009; Hessler et al., 2012; Wang et al., 2017). Bacterial cells acquire this protection from EPS *via* two main mechanisms. First, EPS is a physical barrier between environmental stressors and cell membrane. For example, it was found that TiO_2_ nanoparticles were retained in the EPS and could not directly interact with the cell membrane (Hessler et al., 2012). Second, environmental contaminants could chemically interact with EPS through many EPS-associated functional groups (e.g., carboxyl, amine, hydroxyl, and phosphoric groups). In fact, microbial EPS could reduce metal ions (e.g., Ag^+^ and Au^3+^) to elemental nanoparticles through hemiacetal groups, resulting in decreased toxicity from metal ions (Kang et al., 2014, 2017). Therefore, EPS could probably hinder the LGT, which is vital to the transfer of ARGs in the environment, but has rarely been explored.

Possible intriguing relationships between the abundance of bacterial resistome (ARB and ARGs) and bacterial EPS were discussed in two recent studies. Zhu et al. (2017) found that EPS (especially proteins and polysaccharides) can retain free ARGs to reduce the abundance of ARGs in effluents from membrane bioreactors. Furthermore, after removing EPS from biofilm, Wang et al. (2017) observed a general decline of bacterial abundance at the genus level in the EPS-free biofilm upon the exposure to sulfamethizole. They concluded that EPS may adsorb sulfamethizole and thus decrease the biocidal effect of sulfamethizole, whereas the removal of EPS enhanced the penetration of sulfamethizole into the biofilm and subsequently decreased the bacterial abundance. Nonetheless, these earlier studies did not examine how EPS could influence the lateral transfer of extracellular genes that are released from lysed cells. To our best knowledge, little work has been undertaken to elucidate the effect of EPS on the lateral transfer of extracellular ARGs, which was the focal point of this study.

Therefore, this study aimed to explore the function of EPS in controlling the lateral transfer of ARGs carried by antibiotic resistance plasmids (ARPs) to Ca^2+^-induced competent bacteria cells as ARGs are frequently located in plasmids (Mao et al., 2014). Many studies reported that calcium ion (Ca^2+^) ubiquitously present in the environment can induce natural competence of bacteria (Baur et al., 1996; Hisano et al., 2014; Traglia et al., 2016). *Escherichia coli DH5α* and *XL1 Blue* were chosen as model bacteria because they are highly transformable. The transformation potential of these two strains was adjusted to the optimal using a Ca^2+^ concentration of 0.05 mol L^-1^ that was greater than typical Ca^2+^ concentrations in the environment but was needed to achieve a greater sensitivity in measuring the effect of EPS on the bacterial transformation (Baur et al., 1996). Plasmids (pUC19, pHSG298, and pHSG396), respectively, carrying ampicillin, kanamycin, and chloramphenicol resistance genes were used. The lateral transfer efficiencies of these ARGs into *E. coli DH5a* and *XL1 Blue* cells with and without EPS were determined, which were then corroborated with measurements on cell permeability with and without EPS and binding between EPS and plasmids in the presence and absence of Ca^2+^. Possible interaction sites among plasmids, EPS, and Ca^2+^ were probed by X-ray photoelectron spectroscopy (XPS). Computational modeling was utilized to determine the types and intensity of molecular attractions among Ca^2+^, plasmids, and EPS and to explain their binding mechanisms. This study elucidated the function of EPS in controlling the lateral transfer of extracellular ARGs in bacteria on the molecular and cellular scales and highlighted the vital role of EPS as a means for bacteria to adapt and respond to environmental changes. Its results could help better understand and mitigate the proliferation of ARGs in natural or engineered systems such as soils, sediments, and engineered bioreactors.

## Materials and Methods

### Materials

Three plasmid DNA (pUC19, pHSG298, and pHSG396) were purchased from Takara Biotechnology (China) Co., Ltd. The plasmids were suspended in the solution of 10 mM Tris–HCl (pH 8.0) and 1 mM EDTA at a concentration of 0.5 μg μl^-1^. pUC19 (2686 bp), pHSG298 (2675 bp), and pHSG396 (2238 bp) carry ampicillin, kanamycin, and chloramphenicol resistance genes, respectively (Supplementary Table S1). NaCl, tryptone, and yeast extract in biotechnology grade were purchased from Oxoid (England) Co, Ltd. Agar powder, ampicillin sodium salt, kanamycin, and chloramphenicol were purchased from Solarbio Science and Technology (China) Co., Ltd. CaCl_2_⋅2H_2_O and propidium iodide were purchased from Sigma–Aldrich (St. Louis, MO, United States).

### Bacteria Culture

The Gram-negative *E. coli DH5α* and *XL1 Blue* strains were separately inoculated to the liquid LB culture (10 g l^-1^ of tryptone, 5 g l^-1^ of yeast extract, and 10 g l^-1^ of NaCl, pH 7.0). After shaking at 150 r min^-1^ and 37°C for 12 h, it was transferred into the liquid LB culture at a 1% volume ratio and was then cultivated under the same condition until the bacteria reached the logarithmic growth phase (OD_600_ = 0.4–0.6).

### EPS Extraction

Extracellular polymeric substances were extracted from the *E. coli DH5α* and *XL1 Blue* cells using the method described in the literature (Comte et al., 2006). The suspension was centrifuged at 3000 × *g* and 4°C for 10 min to separate cells from the liquid LB. The bacterial cells were repeatedly washed and then suspended to 20% of the original volume in ultrapure water. After that, the suspension was sonicated at an intensity of 2.7 W⋅cm^2^ and a frequency of 40 Hz at 4°C for 10 min to separate the EPS from the cells and then centrifuged at 10,600 × *g* at 4°C for 20 min. The supernatant was collected and filtered through a microfiltration membrane (0.45 μm) to remove unsettled cells. The obtained pellets and the filtrate were stored at 4°C for the next step. The composition of the EPS solution was determined using the methods described in the Supplementary Information. Total organic carbon (TOC) concentration (after about 4 h of cultivation) was 1.73 and 2.84 mg L^-1^, protein content (by dry weight) was 100 and 97.6 mg g^-1^, and carbohydrate content (by dry weight) was 306 and 311 mg g^-1^ for *E. coli DH5α* and *E. coli XL1 Blue*, respectively.

### Preparation of Competent Cells and Transformation Experiments

**FIGURE 1:**
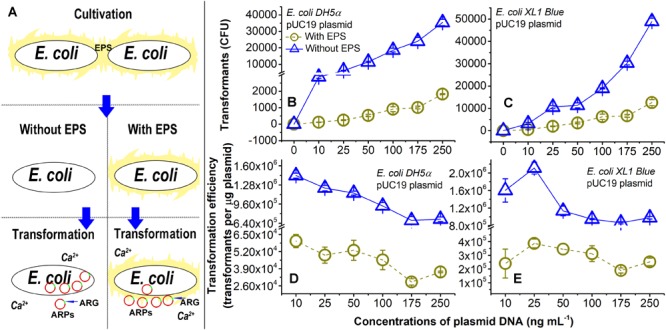
Lateral transfer of pUC19 plasmid into Ca^2+^-induced competent *E. coli* cells with extracellular polymeric substances (EPS; circles) or without EPS (triangles) at pH 7.0. **(A)** Brief introduction of experimental procedures. **(B)** Number of transformants for the lateral transfer of pUC19 into *E. coli DH5α*. **(C)** Number of transformants for lateral transfer of pUC19 into *E. coli XL1 Blue*. **(D)** Transformation efficiency for lateral transfer of pUC19 into *E. coli DH5α*. Transformation efficiency was calculated as transformants per microgram of plasmids. **(E)** Transformation efficiency for lateral transfer of pUC19 into *E. coli XL1 Blue*. Error bars represent standard deviations of triplicates.

The brief experimental procedure is shown in Figure 1A. Briefly, the *E. coli* cells without EPS were obtained from the previous step. The cells with EPS were only washed several times to remove the residual LB solution. Both the *E. coli* cells with and without EPS were suspended in a pre-cooled CaCl_2_ solution (0.05 mol L^-1^) and then placed in an ice-water bath for 30 min. After centrifugation at 3000 × *g* and 4°C for 10 min, the supernatant was removed, and the cell pellets were again suspended in the CaCl_2_ solution (0.05 mol L^-1^).

In the transformation experiments, working solutions of each plasmid DNA (pUC19, pHSG298, or pHSG396) were prepared at various concentrations by diluting the stock solution of 0.5 μg μl^-1^ with Milli-Q water (18 MΩ⋅cm; Millipore, Billerica, MA, United States). Then, 10 μl of each plasmid working solution was added into the suspensions of each competent strain to obtain the final plasmid concentration of 10, 25, 50, 100, 175, or 250 ng ml^-1^. Each treatment was prepared in triplicate. All tubes were shaken gently and placed in the ice-water bath for 30 min. Afterward, the mixture was heat-shocked at 42°C for 90 s and then immediately placed in the ice-water bath for 90 s. Then, the liquid LB media were added to the mixture to result in a total culturing volume of 1 ml, and all tubes were shaken at 150 r min^-1^ and 37°C for 1 h.

To examine the transformation, a bacteria culture of 100 μl was spread on the solid LB medium containing corresponding antibiotics (i.e., 100 mg l^-1^ of ampicillin sodium for pUC19, 50 mg l^-1^ of kanamycin for pHSG298, and 170 mg l^-1^ of chloramphenicol for pHSG396). After 30 min, the plates were inverted and incubated at 37°C for 20 h. Finally, the number of bacterial colonies was counted to determine the transformant number, and the transformation efficiency was calculated as follows:

(1) $$\begin{array}{c} \mathrm{Transformation}\;\mathrm{efficiency}\;=\;\frac{\mathrm{Transformants}\;(\mathrm{CFU})}{\mathrm{The}\;\mathrm{amount}\;\mathrm{of}\;\mathrm{added}\;\mathrm{plasmid}\;(\mu \;g)} \end{array}$$

where transformants refer to 10 times of the bacterial colony number growing on the plates (i.e., the number of transformants in the LB culture).

### Cell Permeability With or Without Extracellular Polymeric Substances

It is known that sufficient cell membrane permeability is necessary for an efficient lateral transfer of ARGs (Bureau et al., 2000). Therefore, to clarify the mechanisms for the effect of the EPS removal on the transfer of ARPs into *E. coli*, we measured cell permeability using the propidium iodide dye staining method. In this method, propidium iodide is absorbed by bacterial cells and then bound to nuclear DNA, leading to the emission of fluorescence that can be quantified by flow cytometry (Haberl et al., 2010). Measured fluorescence intensity is proportional to cell permeability as propidium iodide needs to pass through cell membrane before binding to nuclear DNA (Haberl et al., 2010). Briefly, the suspensions of cells with or without EPS in the presence or absence of Ca^2+^ were spiked with 10 μl of propidium iodide solution (1 μg ml^-1^) and then incubated at 25°C in the dark for 30 min. Afterward, red fluorescence and side scatter emitted from the cells were measured by flow cytometry (BD Accuri C6) at an excitation wavelength of 488 nm. In each run, 20,000 cells were measured in total. The data were then analyzed by the Flowjo software.

### Fluorescence Quenching Titration

Fluorescence quenching titration (Mcintyre and Guéguen, 2013) was employed to determine the binding between plasmids and EPS, in the presence or absence of Ca^2+^. For the binding of plasmids with EPS in the presence of Ca^2+^, the EPS solution extracted from *E. coli* cells was supplemented with the CaCl_2_ solution and sterile Milli-Q water to obtain the final Ca^2+^ and the EPS concentration of 0.05 mol l^-1^ and about 40 mg C l^-1^ (represented by TOC), respectively. The plasmid solution (the quencher) was diluted into a concentration of 2.5 ng μl^-1^. For the binding of plasmids with EPS in the absence of Ca^2+^, the EPS solution was supplemented only with sterile Milli-Q water to obtain the same final EPS concentration of about 40 mg C l^-1^. The plasmid solution was diluted into a concentration of 50 ng μl^-1^. The greater plasmid concentration had to be used because the plasmid solution of lower concentrations showed no apparent fluorescence quenching effect in our preliminary test. For the quenching titration, the plasmid solution was gradually titrated into 20 ml of EPS solution, and then the mixture was fully equilibrated for 20 min at 160 r min^-1^, pH 7.0, and room temperature (25°C) by using a magnetic stirrer. Finally, the fluorescence spectra and intensities were obtained at the 200–310 nm excitation wavelength and the 250–500 nm emission wavelength.

The Stern–Volmer equation describing the fluorescence intensities as a function of plasmid concentrations was used to characterize the interaction between plasmids and EPS:

(2) $$\begin{array}{c} \frac{F_{0}}{F}=1+K_{q}\;\tau_{0}[Q]=1+K_{SV}[Q] \end{array}$$

where *F*_0_ and *F* are fluorescence intensities for the EPS before and after mixing with the quencher, *K*_q_ is the bimolecular quenching rate constant, *τ*_0_ is the average lifetime of the fluorophore before quenching, [*Q*] is the concentration of quencher in the solution, and *K*_SV_ is the Stern–Volmer quenching constant. For the static quenching, the association constant (*K*_A_) can be calculated via

(3) $$\begin{array}{c} Log(\frac{F_{0}-F}{F})=LogK_{A}+nLog[Q] \end{array}$$

### Spectroscopic Analyses

To identify the functional groups of EPS before and after reaction with DNA and Ca^2+^, XPS analyses were performed. Ten milliliters of EPS–Ca^2+^–DNA solution was prepared and incubated at 25°C for 2 h for a thorough reaction. Meanwhile, the pristine EPS solution and EPS–Ca^2+^ solution were also similarly prepared. After freeze-drying, the XPS spectra of all the samples were collected using an ESCALAB 250Xi (Thermo Scientific, United States) with a monochromatic Al Ka X-ray source.

### Computational Modeling

The molecular modeling was performed to further explore the binding mechanisms among EPS, Ca^2+^, and plasmid DNA. Since the composition of EPS is extremely complex, we chose to use five amino acids as the smallest unit of proteins and five monosaccharides as the smallest units of polysaccharides in the representation of EPS. Also, according to the XPS results discussed later, the phosphate group was selected here to represent the reactive site in the plasmid DNA. Although DNA bases could also be involved in the interaction, we focused on the DNA phosphate backbone as the main reactive site since Ca^2+^ was reported to facilitate enzyme (protein)–DNA binding mainly through the phosphate backbone (Bellamy et al., 2009). Moreover, the binding of protein or polysaccharide to DNA bases only occurs under certain conditions; i.e., proteins bind to the DNA bases mainly *via* specific protein–DNA interaction involving base pairs of the cognate operator sequence (Kalodimos et al., 2004), and polysaccharides bind to DNA bases at high concentrations (Tajmir-Riahi et al., 1994). Thus, the interaction between EPS and DNA bases is expected to be very limited. A detailed description of the molecular computation is provided in the Supplementary Information.

## Results and Discussion

### Lateral Transfer of ARGs in Plasmids

As shown in Figure 1B,C, the number of transformants increased with increasing concentrations of pUC19 (0–250 ng ml^-1^) regardless of the EPS removal for *E. coli DH5α* and *E. coli XL1 Blue*. The transformation efficiencies (Figure 1D,E) were the greatest at the plasmid concentration of 10 ng ml^-1^ for *E. coli DH5a* and 25 ng ml^-1^ for *E. coli XL1 Blue*. There were varying trends in the transformation efficiency as a function of plasmid concentrations (Figure 1 and Supplementary Figure S1). It is noted that the three plasmids had very different transformation efficiencies for even the same *E. coli* strain (Figure 1D,E and Supplementary Figures S1E–H). The difference may be due to variations in sizes (Lu et al., 2012) or specific sequences (Edman, 1992) of plasmids. For the plasmids with sizes ranging from 2 to ∼3.5 kbp, the transformation efficiency increased with increasing the plasmid size (Lu et al., 2012). Thus, pUC19 (2686 bp) and pSHG298 (2675 bp) had greater transformation efficiencies than pSHG396 (2238 bp). However, for pUC19 and pSHG298 with similar plasmid sizes, the difference in their specific DNA sequence might lead to different transformation efficiencies. There was also varying transformation efficiency for the same plasmid between *E. coli DH5α* and *E. coli XL1 Blue* (Figure 1 and Supplementary Figure S1). Thus, factors influencing transformation efficiency are not only specific to plasmid characteristics but also specific to bacteria type. For example, the lipopolysaccharide in the cell membrane (Panja et al., 2008) or some special genes (e.g., *crp* genotypes; Umemoto et al., 1996) in the bacterial genome could affect the transformation efficiency. Nonetheless, the exact underlying mechanisms for the varying transformation efficiencies in diverse experimental conditions (e.g., plasmid concentration, plasmid type, or bacteria type) remain elusive.

More interestingly, the removal of EPS substantially enhanced the transfer of pUC19 into the *E. coli* cells. For example, the transformation efficiency and the number of transformants at 250 ng ml^-1^ of pUC19 for *E. coli DH5α* and *XL1 Blue* without EPS was nearly 19 and 4 times of those with EPS, respectively. As the lateral transfer of pUC19 into *E. coli DH5α* was often studied (Rivas et al., 2013; Chatterjee and Sarkar, 2014), the transformation efficiency of pUC19 measured in this study agreed with a typical level of 1 × 10^4^ transformants per microgram of plasmid in the presence of the same Ca^2+^ concentration (Lim et al., 2015). When the EPS were removed, the transformation efficiencies increased up to the order of 1 × 10^6^ transformants per microgram of plasmid, indicating a large LGT enhancement due to the EPS removal. For pHSG298 and pHSG396, the transformation efficiency and the number of transformants of *E. coli DH5α* without EPS were nearly 29 and 20 times of that with EPS, respectively (Supplementary Figures S1A,B,E,F). Similarly, the transformation efficiency and the number of transformants for *E. coli XL1 Blue* without EPS was 7 and 4 times of that with EPS for pHSG298 and pHSG396, respectively (Supplementary Figures S1C,D,G,H). These results demonstrated that the removal of EPS substantially increased the transfer of ARPs into the Ca^2+^-induced competent cells, and the EPS played a key role in the LGT of plasmid-borne ARGs. Next, we will further elucidate the underlying mechanisms for the abovementioned observations.

### Cell Permeability Measurements

**FIGURE 2:**
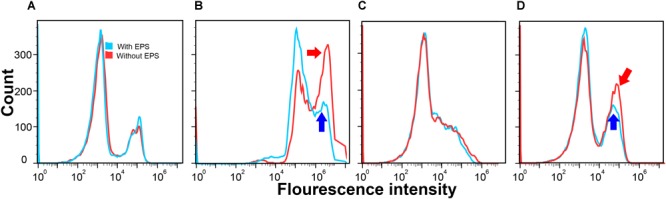
Cell permeability of *E. coli DH5α* **(A,B)** and *E. coli XL1 Blue* **(C,D)** with EPS (blue) or without EPS (red) at pH 7.0 and 30-min incubation time in the absence of Ca^2+^ **(A,C)** and in the presence of Ca^2+^ **(B,D)**.

Figure 2 presents the number of cells (*y*-axis) that emitted certain fluorescence intensity at the *x*-axis. If bacterial cells had greater cell permeability, most of such bacteria cells would have a higher fluorescence intensity, resulting in a cell count peak located toward the right of the *x* axis. As shown in Figure 2A, in the absence of Ca^2+^, the curves of *E. coli DH5α* with (blue) or without (red) EPS were almost the same. Similar results were also found in *E. coli XL1 Blue* in the absence of Ca^2+^ (Figure 2C). This suggests that when the cells lacked the Ca^2+^-induced competence, the removal of EPS did not significantly change the cell permeability. In contrast, in the presence of Ca^2+^ (Figure 2B,D), despite a slight difference in the responses of two strains, the cell permeability was greater than that without Ca^2+^. For *E. coli DH5α*, both peaks shifted toward higher fluorescence intensities compared with those in the absence of Ca^2+^ (Figure 2A). For *E. coli XL1 Blue*, additional peaks (pointed by blue and red arrows) emerged and were located at higher fluorescence intensities relative to that in the absence of Ca^2+^ (Figure 2C). Clearly, Ca^2+^ could enhance the cell permeability, likely due to their effect on the reorganization of membrane lipid bilayer as previously reported (Cambrea et al., 2007).

After removing EPS in the presence of Ca^2+^, for *E. coli DH5α* (Figure 2B) the peak for the cells with EPS (pointed by the blue arrow) located at a higher fluorescence intensity (around 10^6^) had around 150 counts, whereas the peak for the cells without EPS (pointed by the red arrow) had a much higher count (nearly 325 counts). For *E. coli XL1 Blue* (Figure 2D), the additional peak with a higher fluorescence intensity induced by Ca^2+^ for the cells with EPS (pointed by the blue arrow) had around 125 counts. However, the peak for the cells without EPS (pointed by the red arrow) had nearly 200 counts. These results revealed that the EPS removal allowed more *E. coli* cells to absorb more propidium iodide dye, indicating an increased cell permeability. It was reported that a higher cell permeability would result in a higher transfer of pEGFP-N_t_ plasmid into *E. coli DH5α* in a gene electrotransfer experiment at otherwise same conditions (e.g., Mg^2+^ concentrations or electric field strengths; Haberl et al., 2010). Therefore, the enhanced cell permeability would allow more ARPs (pUC19, pHSG298, and pHSG396) to enter into the *E. coli DH5α* and *XL1 Blue* cells, leading to greater transformation efficiencies after the EPS removal.

Moreover, compared to the changes in the cell permeability after the EPS removal between the two *E. coli* strains, the enhancement of cell permeability for *E. coli DH5α* (i.e., about 175 more cell counts) appeared greater than that of *E. coli XL1 Blue* (nearly 75 more cell counts). It is possible that the EPS of the two *E. coli* strains might have different capacities to retain Ca^2+^, as demonstrated in the fluorescence titration discussed next. As a result, the removal of EPS would have different effects on the amount of Ca^2+^ that could bind with lipopolysaccharides in the cell membrane, resulting in the varying enhancement of cell permeability as reported by previous studies (Haberl et al., 2010). This trend also corroborated with the LGT results that the removal of EPS led to a greater transfer of plasmid-borne ARGs into *E. coli DH5α* than into *E. coli XL1 Blue*. In other words, the increased lateral ARG transfer without EPS was due to the enhanced cell permeability after the EPS removal.

### Binding of ARPs With EPS

It is hypothesized that bacterial EPS could serve as a barrier to the entry of ARPs into bacterial cells, and thus the removal of EPS could cause an increased transfer of ARPs. To test this hypothesis, we determined the binding of plasmids with EPS in the absence or presence of Ca^2+^ by the fluorescence quenching experiments. Only one fluorescence peak exists in the three-dimensional fluorescence excitation–emission matrix (EEM) spectra for the two *E. coli* strains (excitation/emission = 285/340 nm; Supplementary Figure S2), and this peak is ascribed to the tryptophan fluorophore (Supplementary Table S2). Since other fluorophores were not detectable, tryptophan may be used as a fluorescence probe for the EPS of the two strains.

**FIGURE 3:**
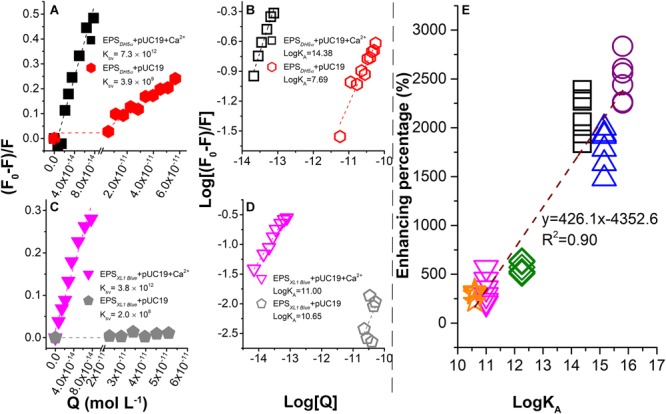
Binding between plasmids and EPS in the presence or absence of Ca^2+^ probed by fluorescence quenching at 25°C and pH 7.0. Stern–Volmer plots for *E. coli DH5α* **(A)** and *E. coli XL1 Blue* **(C)**. Plots of log [(*F*_0_ -*F*)/*F*] vs. log [*Q*] for *E. coli DH5α* **(B)** and *E. coli XL1 Blue* **(D)**. **(E)** Correlation between LGT enhancement percentage (%) due to the EPS removal and association constants (Log*K*_A_) between plasmids and EPS in the presence of Ca^2+^. The data for *DH5α* + pSHG298, *DH5α* + pSHG396, *XL1 Blue* + pSHG298, and *XL1 Blue* + pSHG396 are shown in the Supplementary Information.

From the linear fitting of the Stern–Volmer plots, the *K*_SV_ values in the presence of Ca^2+^ were about 10^3^–10^4^ times of that in the absence of Ca^2+^, suggesting that plasmids could quench the fluorescence of EPS more efficiently in the presence of Ca^2+^ (Figure 3A,C). Since the average fluorescent lifetime (τ_0_) of tryptophan-related chromophores in EPS is nearly 5 × 10^-9^ s, the bimolecular quenching rate constants (*K*_q_) could be calculated as 1.46 × 10^21^ and 7.8 × 10^17^ l mol^-1^ s^-1^ for the EPS of *E. coli DH5α* with or without Ca^2+^, respectively. Similarly, the *K*_q_ values were 7.6 × 10^20^ and 4 × 10^16^ l mol^-1^ s^-1^ for the EPS of *E. coli XL1 Blue* with or without Ca^2+^, respectively. These *K*_q_ values are much greater than the maximum *K*_q_ value for a diffusion-controlled quenching of a biopolymer (nearly 2.0 × 10^10^ l mol^-L^ s^-s^; Lakowicz, 1983). Therefore, the fluorescence quenching of EPS by plasmids in the presence or absence of Ca^2+^ could all result from the static quenching, indicating the formation of the plasmid–EPS complex.

The static quenching was further demonstrated in Figure 3B,D with the association constant (Log*K*_A_) representing the binding strength (Qin et al., 2018). In the absence of Ca^2+^, the Log*K*_A_ values for *E. coli DH5α* and *E. coli XL1 Blue* were 7.69 and 10.65 l mol^-1^, whereas in the presence of Ca^2+^, the Log*K*_A_ values were increased to 14.38 and 11 l mol^-1^, respectively. Therefore, Ca^2+^ significantly strengthened the binding between the plasmids and EPS. A previous study (Sheng et al., 2013) reported that Ca^2+^ had no significant quenching capacity for EPS as it was thought that Ca^2+^ did not form a chemical bond with EPS. However, the addition of Ca^2+^ made the zeta potential of EPS substantially more positive, suggesting that Ca^2+^ could be sorbed by EPS through electrostatic attraction and thus neutralized the negative surface charge of EPS (Sheng et al., 2013). As a result, the binding of highly negatively charged plasmids to EPS was enhanced.

In the presence of Ca^2+^, Log*K*_A_ for *E. coli DH5α* was higher than that for *E. coli XL1 Blue*. This observation supported the results in Figure 1B–E, showing that the EPS removal caused a much greater increase in the ARG transfer into *E. coli DH5α* than into *E. coli XL1 Blue*. To further explore the relationship between the binding strength and transformation enhancement due to the EPS removal, the enhancement percentage (%) as a function of Log*K*_A_ was examined (Figure 3E). The enhancement percentage was calculated as follows:

(4) $$\begin{array}{c} \mathrm{Enhancement}\;\mathrm{percentage}\;(\%)\;=\;\frac{\left({A-A}_{0}\right)}{A_{0}}\;\times \;100\% \end{array}$$

where *A*_0_ is the transformation efficiency with EPS and *A* is the transformation efficiency without EPS. The data in Figure 3E were collected from the transformation experiments of pUC19 (Figure 1D,E), pSHG298 (Supplementary Figures S1E,G), and pSHG396 (Supplementary Figures S1F,H) at concentrations of 10–250 ng ml^-1^, and the fluorescence quenching experiments for the EPS of two *E. coli* strains by these three plasmids (Figure 3B,D and Supplementary Figures S3E–H). A significant positive linear correlation between the enhancement percentage and the Log*K*_A_ values was observed in Figure 3E, indicating that a higher binding strength of plasmids with EPS corresponded to a greater enhancement of the ARG transfer into the bacterial cells with the EPS removal. This is another line of evidence that the bacterial EPS could trap extracellular plasmids and thus hinder the transfer of ARGs carried by the ARPs.

Although the binding of EPS with DNA was rarely reported, the main components of EPS (e.g., protein and polysaccharide) were found to bind to DNA in some studies (Tajmir-Riahi et al., 1994; Kalodimos et al., 2004). For non-sequence-specific protein–DNA interaction, proteins prefer to bind with the DNA backbone mainly through electrostatic or H-bonding interaction (Kalodimos et al., 2004). Sugar moieties (e.g., glucose, fructose, and galactose) in polysaccharides tend to bind with the DNA backbone *via* H-bonding (Tajmir-Riahi et al., 1994). Similarly, the EPS interaction with DNA in the presence of divalent ions was also rarely studied. Ca^2+^ and Mg^2+^ are well-known catalytic cofactors for enzymatic action on DNA due to the facilitation of enzyme–DNA binding by Ca^2+^ and Mg^2+^. Moreover, it was reported that Ca^2+^ could interact with carbohydrate (polysaccharides; Siuzdak et al., 1993) and DNA, respectively, which might allow Ca^2+^ to bridge polysaccharides and DNA. As a result, the negative-charged sites of amphoteric EPS could also bind to DNA *via* cation bridging, which was supported by the fact that the presence of Ca^2+^ facilitated the binding of EPS with plasmids in the study. As Ca^2+^ is ubiquitous in the environment, the induction of bacterial cell competence by Ca^2+^ would facilitate the transfer of ARPs to bacterial cells. However, as a counterbalance, Ca^2+^ could enhance the trapping of ARPs by EPS and thus offset the enhancement of the ARP transfer by Ca^2+^. As such, the role of EPS in hindering the ARP (ARG) transfer may become particularly important in Ca^2+^-enriched environments such as calcareous soils, sediments, and waters.

### Binding Mechanism of ARPs With EPS

Despite the perceived important ecological function of DNA binding with EPS on the LGT, the detailed binding mechanisms, including possible binding sites and binding strength, were still little known. Here, we used XPS spectra and model computation to elucidate the binding mechanisms of plasmid DNA with EPS in the absence or presence of Ca^2+^.

**FIGURE 4:**
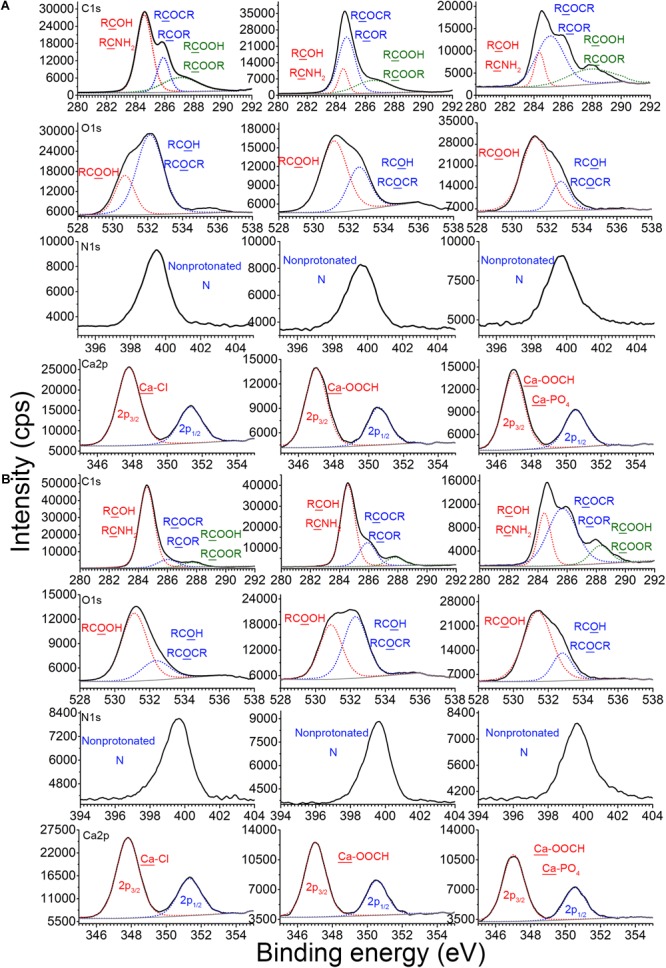
Binding sites of EPS with plasmid DNA in the absence or presence of Ca^2+^ at pH 7.0 and 25°C. X-ray photoelectron spectroscopy (XPS) analysis of C, N, and O of EPS before and after reaction with plasmids in the absence or presence of Ca^2+^, and XPS analysis of Ca in CaCl_2_, Ca^2+^–EPS, and Ca^2+^–EPS–DNA, for *E. coli DH5α* **(A)** and *E. coli XL1 Blue* **(B)**.

X-ray photoelectron spectroscopy analysis was used to explore the binding sites among EPS, plasmid, and Ca^2+^. Figure 4A shows the XPS spectra of the EPS of *E. coli DH5α* before and after reaction with pUC19 in the absence or presence of Ca^2+^. C1s peaks of CNH_2_ or RCOH (at around 284.6 eV), RCOCR or RCOR (at around 286.0 eV), and RCOOH (at around 287.2 eV) for pristine EPS were shifted to 284.4, 284.6, and 286.8 eV after reaction with pUC19, respectively. The O1s peaks of RCOH or RCOCR (at around 532.2 eV) and RCOOH (at around 530.8 eV) were also shifted to 532.6 and 531.2 eV, respectively. These shifts indicate that plasmid DNA might bind with the EPS of *E. coli DH5α* through hydroxyl or amino groups, glucoside, hemiacetal, and carboxylate (Omoike and Chorover, 2004). In the presence of Ca^2+^, the C1s peak of COOH, the O1s peak of COOH, and the N1s peak of non-protonated N were further shifted by 1.6, 0.2, and 0.4 eV, respectively, compared to the peaks in the absence of Ca^2+^ (Figure 4A), suggesting that major interaction sites of Ca^2+^ might locate at the carboxyl group of protein in EPS. This agreed with a previous study reporting that Ca^2+^ could bind to amino acids due to the electrostatic interaction with the carboxyl group (Rhilassi et al., 2012). Hydroxyl group and glucoside could be other interaction sites of Ca^2+^ with EPS since O1s peaks of RCOH or RCOCR and the C atom in RCOCR of glucoside were shifted further compared to the shift in the absence of Ca^2+^.

For *E. coli XL1 Blue* (Figure 4B), the binding sites of EPS for the plasmids in the absence of Ca^2+^ might be mainly located at the carboxyl group in the protein. Since after the EPS binding with plasmids, only the peak of COOH was shifted from 581.2 to 580.0 eV. In the presence of Ca^2+^, these peaks for the carboxyl group were further shifted and strengthened to a larger extent, e.g., the peaks of COOH were shifted to 581.4 eV (Figure 4B). In addition, the O1s peaks of RCOH or RCOCR were at 532.4 eV when interacting with plasmids, but were shifted to 532.8 eV in the presence of Ca^2+^. Thus, in addition to interaction with the carboxyl group of proteins, Ca^2+^ could also interact with the EPS of *E. coli XL1 Blue* through the hydroxyl group and glucoside of polysaccharides, similar to *E. coli DH5α*.

Additionally, the Ca2p_3/2_ peak of CaCl_2_ was located at 348.0 eV, typical of the binding energy for Ca–Cl (Demri and Muster, 1995). After reaction with the EPS of *E. coli DH5α* and *E. coli XL1 Blue*, the Ca2p_3/2_ peaks were both shifted to 347.2 eV (the representative binding energy for Ca–OOCH; Demri and Muster, 1995), suggesting that Ca^2+^ bound to EPS through the carboxyl group. In the presence of Ca^2+^, EPS, and plasmid, the Ca2p_3/2_ peaks were further shifted nearly to 370.0 eV (typical binding energy for Ca–PO_4_; Demri and Muster, 1995) for both strains. Therefore, in the presence of plasmids and EPS, in addition to binding to the carboxyl group of EPS, Ca^2+^ also bound to the phosphate backbone of plasmid, which indisputably confirmed Ca^2+^ bridging of the plasmids and EPS.

**FIGURE 5:**
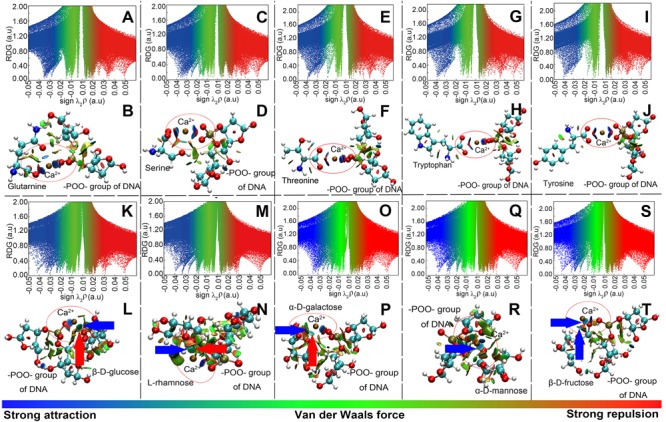
Model computation of the interaction of the –POO– group of DNA with amino acids (representing proteins in EPS; **A–J**) or with monosaccharides (representing polysaccharides in EPS; **K–T**), in the presence of Ca^2+^. **(A,C,E,G,I,K,M,O,Q,S)** Plots of the reduced density gradient versus the electron density multiplied by the sign of the second Hessian eigenvalues. **(B,D,F,H,J,L,N,P,R,T)** Gradient isosurfaces. **A** and **B** were for the glutamine; **C** and **D** were for the serine; **E** and **F** were for the threonine; **G** and **H** were for the tryptophan; **I** and **J** were for the tyrosine; **K** and **L** were for the glucose; **M** and **N** were for the rhamnose; **O** and **P** were for the galactose; **Q** and **R** were for the mannose; **S** and **T** were for the fructose. Atoms **C, O, N, P**, and **H** were labeled by green, red, blue, brown, and white, respectively. Solvent was taken into consideration.

Model computation was employed to show the intensity and types of molecular attractions among DNA, EPS, and Ca^2+^, as represented by the color of gradient isosurfaces in Figure 5. The blue color in the gradient isosurfaces indicates a strong attraction of the two molecules (e.g., coordination interactions or electrostatic interaction), the green refers to van der Waals interactions, and the red indicates a strong repulsion. Accordingly, for the plots of reduced density gradient (RDG) versus the electron density multiplied by the sign of the second Hessian eigenvalues (Figure 5A,C,E,G,I,K,M,O,Q,S), the spikes located at sign(λ)ρ value less than -0.02 a.u. and larger than -0.05 a.u. mean a strong non-covalent attraction and the attraction is stronger with a more negative value. The spikes located at the sign(λ)ρ value between -0.02 and 0.02 a.u. are regarded as van der Waals interactions, and at a value greater than 0.02 a.u., the interaction is repulsive. As shown in the gradient isosurfaces of five amino acids with DNA (Figure 5B,D,F,H,J), the apparent blue color appeared between –POO– in the phosphate backbone and Ca^2+^ and between the C = O in the amino acids and Ca^2+^ (marked by the red circle). Moreover, from Figure 5A,C,E,G,I, the corresponding spikes (with blue color) were all located at more negative than -0.02 a.u. but less negative than -0.05 a.u. (Johnson et al., 2010). This means that Ca^2+^ bridged the plasmids and proteins in EPS through strong non-covalent attractions (likely electrostatic interactions). It was noted that EPS in addition to Ca^2+^, the DNA phosphate backbone and the proteins in EPS could bind with each other *via* relatively weak van der Waals interactions, as shown in Figure 5 [i.e., the green color between the two molecules with corresponding sign(λ)ρ of green spikes > -0.02 a.u]. However, compared to the interaction of Ca^2+^ with DNA or proteins, these interactions are too weak to maintain the tight binding of DNA and proteins. Thus, the lower association constant (Log*K*_A_) values were observed between plasmids and EPS in the absence of Ca^2+^ (Figure 3B,D). Similarly, the interaction between Ca^2+^ and the DNA phosphate backbone (through –POO–) or monosaccharide (pointed by the blue arrow; through C–OH) was relatively stronger [blue color, sign(λ)ρ < -0.02 a.u.; Figure 5K–T]. Moreover, some relatively weak interactions were also observed between RC–O–CR in monosaccharide and Ca^2+^ (pointed by the red arrow), which was in accordance with the results of XPS analysis. Also, almost all the interactions between monosaccharides and DNA were attributed to weak van der Waals forces. Thus, the computational evidence proved that Ca^2+^ could strongly link the plasmids and polysaccharides together.

## Conclusion

The spread of ARGs in the environment has raised serious concerns, since it can increase the risk of the lateral ARG transfer to human pathogens and thus pose enormous threats to human health. By examining the transfer of plasmid-borne ARGs into Ca^2+^-induced competent *E. coli DH5α* and *XL1 Blue* with and without EPS, this study examined the role and function of EPS in the lateral ARG transfer on the molecular and cellular scales. It was found that bacterial cells without EPS had greater transformation efficiencies than those with EPS. The underlying mechanisms include the following: first, the greater cell permeability without EPS in the presence of Ca^2+^ is essential to greater transformation efficiencies; second, EPS could trap the ARG-carrying plasmids facilitated by Ca^2+^ bridging of EPS and plasmids, thus hindering the entry and lateral transfer of ARG into recipient cells.

It might be possible that the EPS removal might enhance the transfer of plasmid-borne ARGs through other potential mechanisms (such as the change in cell viability and cell membrane integrity). In our study, the EPS removal did not influence the viability of bacteria, since the cell survival showed insignificant differences between the cells with and without EPS, as also reported by others (Kang et al., 2017). Therefore, the ARG transfer enhancement induced by the EPS removal should not be attributed to the change in cell viability. Under certain conditions, some tiny holes may exist on the cell membrane due to the oxidative stress response (SOS response) of bacteria to external stress, which could benefit the genetic transfer of ARG (Qiu et al., 2012). Nevertheless, the bacterial cell membrane after the EPS removal was relatively intact in this study, since no membrane damage was reported for the *E. coli* without EPS (Kang et al., 2014).

Overall, this study provided new insights into the interaction of ARPs with EPS and the function of EPS in the genetic transfer of ARGs in bacterial populations. Nonetheless, this study was limited in terms of the use of *E. coli* strains as model bacteria and relatively simplistic culturing conditions (i.e., aqueous culture). Despite the fact that Gram-negative *E. coli* strains are becoming increasingly persistent in soils, sediments, or wastewaters (Ishii and Sadowsky, 2008; Elsas et al., 2011), they are only pseudo-native to these environmental niches and are primarily native to the intestines of humans and animals. Therefore, future studies should be directed to other types of bacteria more representative in the environment (e.g., the Gram-positive Actinobacteria) and to more realistic experimental conditions mimicking soils, sediments, freshwater, or wastewaters. Other environmental factors (such as the presence of humic substances and biofilm) could also be considered. By extending the knowledge on the molecular and cellular scales observed in this study, future studies should improve our understanding on the function of EPS in regulating the ARG transfer on the ecological scale.

## Author Contributions

XH and YG designed the research. XH performed the experiments. BY and FK analyzed the computational data. XH, BY, and CQ analyzed the experimental data. XH, YG, and WZ wrote the manuscript. All authors contributed to the scientific discussion.

## Conflict of Interest Statement

The authors declare that the research was conducted in the absence of any commercial or financial relationships that could be construed as a potential conflict of interest.
