# Supplementary material for: Extracellular Polymeric Substances Acting as a Permeable Barrier Hinder the Lateral Transfer of Antibiotic Resistance Genes
Source: Front Microbiol. 2019 Apr 17;10:736. doi: 10.3389/fmicb.2019.00736 (PMC6479211; doi:10.3389/fmicb.2019.00736)
Supplement: Supplementary file 1 [file Table_1.docx]

Supplementary Material

# Supplemental methods

## EPS compositions.

Total organic carbon (TOC) of the EPS solution was analyzed using a TOC-5000A analyzer (Shimadzu, Kyoto, Japan). The protein content in the EPS was obtained by the Lowry method using bovine serum albumin as the standard (Frølund et al., 1995). The carbohydrate content was measured by the phenol-sulfuric acid method using glucose as the standard (Dubois et al., 1956). The DNA content was determined by the diphenylamine colorimetric method using calf thymus DNA as the standard (Burton, 1956).

## Model computation.

In accordance with the binding sites for the EPS-Ca^2+^-DNA interaction in the XPS analyses combined with the findings in a previous study (Kang et al., 2013), five amino acid models and five monosaccharide models were structured to represent EPS and -POO- group was used to represent DNA. The geometries were constructed using the ChemBioOffice software, and the Semi-Empirical Method PM7 (Mopac2012 program) was applied to initially optimize these geometries (Stewart). Then, all structural optimization and frequency analyses were carried out by applying the density functional theory (DFT) at a main def2-TZVP basis set that is an auxiliary def2-TZVP/J basis set with dispersion-corrected DFT-D3 and GCP (DFT/SVP) (Neese, 2012). After that, the wave functions obtained in previous step were analyzed by applying the Multiwfn program to get the information of reduced density gradient (RDG). The topological analysis and a graphic illustration of the distribution of the electron density in EPS-Ca^2+^-DNA were also obtained by using the Multiwfn program.

# Supplemental figures and tables





**Figure S1.** Lateral transfer of plasmids into Ca^2+^-induced *E. coli* cells with EPS (circles) or without EPS (triangles). (**A**) Number of transformants for the lateral transfer of pHSG298 into *E. coli DH5α*. (**B**) Number of transformants for the lateral transfer of pHSG396 into *E. coli DH5α*. (**C**) Number of transformants for the lateral transfer of pHSG298 into *E. coli XL1 Blue*. (**D**) Number of transformants for the lateral transfer of pHSG396 into *E. coli XL1 Blue*. (**E**) Transformation efficiency for lateral transfer of pHSG298 into *E. coli DH5α*. The transformation efficiency was calculated as transformants per μg plasmids. (**F**) Transformation efficiency for the lateral transfer of pHSG396 into *E. coli DH5α*. (**G**) Transformation efficiency for the lateral transfer of pHSG298 into *E. coli XL1 Blue*. (**H**) Transformation efficiency for the lateral transfer of pHSG396 into *E. coli XL1 Blue*. Error bars represent standard deviations of triplicates.


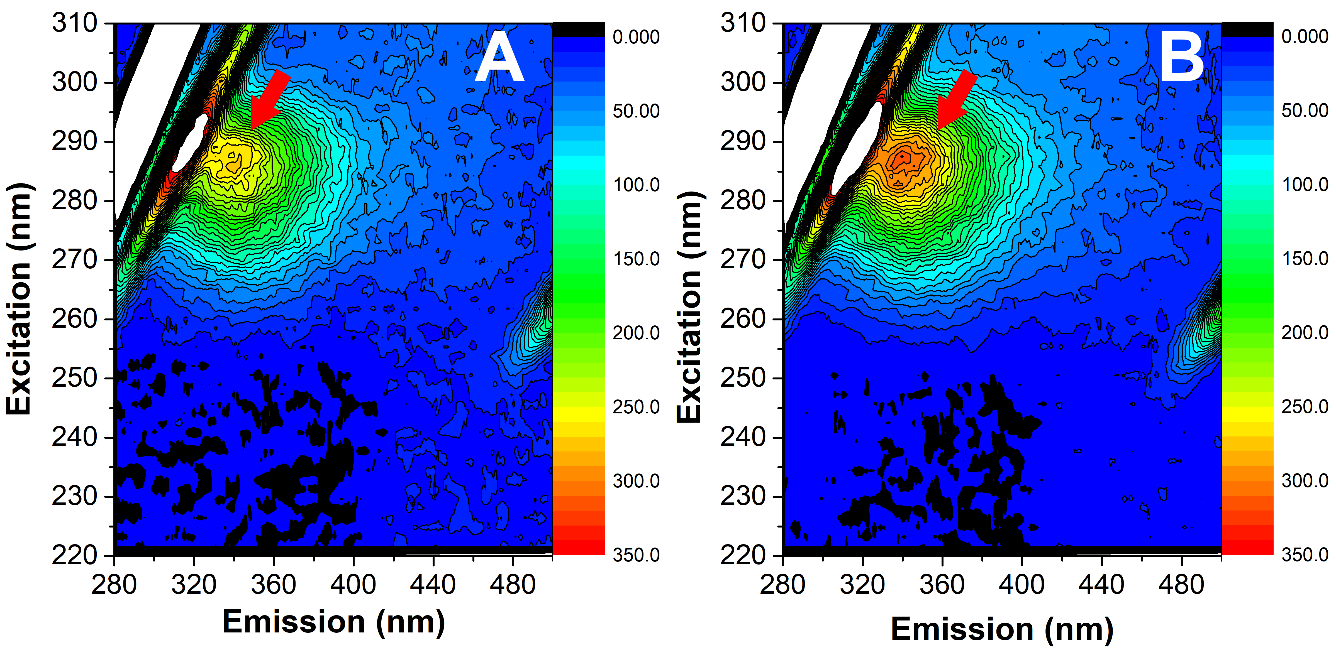


**Figure S2.** Typical three-dimensional fluorescence excitation-emission matrix (EEM) spectra of original EPS for *E. coli DH5α* (**A**) and *E. coli XL1 Blue* (**B**) at pH 7.0 at 25 ℃. The EPS solution extracted from *E. coli* cells was diluted tenfold. The fluorescence EEM spectra were obtained at 200–380 nm excitation wavelength and 250–500 nm emission wavelength.





**Figure S3.** Binding of pHSG298 and pHSG396 with EPS in the presence of Ca^2+^ probed by fluorescence quenching at pH 7.0 at 25 ℃. (**A**-**D**) Stern−Volmer plots for the EPS of *E. coli* *DH5α* quenched by pHSG298 (**A**) and pHSG396 (**B**), and for the EPS of *E. coli XL1 Blue* quenched by pHSG298 (**C**) and pHSG396 (**D**). (**E**-**H**) Plots of log [(F_0_-F)/F] vs. log [Q] for the EPS of *E. coli DH5α* quenched by pHSG298 (**E**) and pHSG396 (**F**), and for the EPS of *E. coli XL1 Blue* quenched by pHSG298 (**G**) and pHSG396 (**H**). Notes: The EPS solution extracted from the *E. coli* cells was filled with CaCl_2_ solution and sterile Milli-Q water to a final Ca^2+^ concentration up to 0.05 mol L^-1^ (Millipore, 18 MΩ·cm) and the EPS concentration was up to nearly 40 mg C L^-1^. The plasmid stock solution was diluted with sterile Milli-Q water to achieve the final plasmid concentration of up to 2.5 ng μL^-1^. The plasmid solution was gradually titrated into 20 mL of the EPS solution and then mixed by magnetic stirring for 20 min at 160 rpm, pH 7.0, and 25 °C. Afterward, the fluorescence spectra and intensities were obtained at 200–380 nm excitation wavelength and 250–500 nm emission wavelength (F96PRO, LengGuang).

**Table S1.** Basic information of plasmids used in this study.

| **Plasmids** | **Antibiotic resistance** | **Chain length**  **(bp)** |
| --- | --- | --- |
| pUC19 | ampicillin resistance | 2686 |
| pHSG298 | kanamycin resistance | 2675 |
| pHSG396 | chloramphenicol resistance | 2238 |

**Table S2.** Peak positions of fluorophores in EPS ([Chen et al., 2003](#_ENREF_1)).

| **Peak** |  | **Fluorescence type** |
| --- | --- | --- |
| **Excitation (nm)** | **Emission (nm)** |  |
| 220 – 250 | 280 – 380 | tyrosine and aromatics in protein-like components |
| > 250 | 280 – 380 | tryptophan in protein-like components |
| 220 – 250 | > 380 | fulvic acid-like components (phenol/quinone structure) |
| > 250 | > 380 | humic acid-like components (phenol/quinone structure) |

**References**

Chen, W., Westerhoff, P., Leenheer, J.A., and Booksh, K. (2003). Fluorescence excitation-emission matrix regional integration to quantify spectra for dissolved organic matter. *Environ. Sci. Technol.* 37:24, 5701-5710. DOI: 10.1021/es034354c

Dubois, M., Gilles, K.A., Hamilton, J.K., Rebers, P.A., and Smith, F. (1956). Colorimetric method for determination of sugars and related substances. *Anal. Chem.* 28:3, 350-356. DOI: 10.1021/ac60111a017

Frølund, B., Griebe, T., and Nielsen, P.H. (1995). Enzymatic activity in the activated-sludge floc matrix. *Appl. Microbiol. Biotechnol.* 43:4, 755-761. DOI: 10.1007/BF00164784

Burton K. (1956). A study of the conditions and mechanism of the diphenylamine reaction for the colorimetric estimation of deoxyribonucleic acid. *Biochem. J.* 62:2, 315-323. DOI: 10.1042/bj0620315

Kang, F., Hong, W., Gao, Y., Jian, L., and Qian, W. (2013). Ca^2+^ Promoted the low transformation efficiency of plasmid DNA exposed to PAH contaminants. *PLoS One* 8:3, e58238. DOI: 10.1371/journal.pone.0058238

Neese, F. (2012). The ORCA program system. *Wiley Interdiscip. Rev.: Comput. Mol. Sci.* 2:1, 73-78. DOI: 10.1002/wcms.81

Stewart, J.J.P. Stewart Computational Chemistry, 15210 Paddington Circle, Colorado Springs, Colorado 80921.
